# Eserine in Diarrhœa

**Published:** 1883-08

**Authors:** 


### Eserine in Diarrhœa.

Dr. Eschle, in the *Neurologisches Centralblatt*, May 15th, reports observations made in the Richter'schen Heilanstalt (Pankow-Berlin) on the curative effects of calabar bean preparations in catarrhal conditions of the digestive tract. Eserine (physostigmin), the alkaloid which with calabarin is found in the seed of the physostigma venenosum, was used some time ago in the same institution, where it gave great satisfaction in quieting maniacal patients, and for such paralytics as were not liable to apopletic attacks. The action of the drug proved similar to, though more lasting and reliable than that of hyoscyamin. The method of exhibition was by the subcutaneous injection of a one-half per cent. solution of sulphate of eserine in doses of .001 gramme to .0015 gramme. Its use was found to be always attended by alteration in the digestive organs. One maniacal patient who cried out continuously was quieted by the use of .0025 gramme; this rather large injection caused vomiting and free watery stools. The use of smaller doses quieted the physical and motor restlessness, and produced sleep without vomiting or defecation. In three other patients (paralytics) stoppage was noted for over thirty-six hours, vomiting occurring only once. Quiet in bed was always ordered. The special object of the communication was to report the results of the use of eserine in three cases of intestinal catarrh.

The first patient suffered from an attack of this nature, causing continual desire to defecate, with a passage every half hour during the second day. Hypodermic injection on this day of .001 gramme of the eserine solution produced sleep in an hour and a half, which lasted from two o'clock till evening. No passage occurred until forty-six hours after the injection.

In the second case intestinal catarrh was brought on by a cold. A large number of watery stools were passed during the night and on the following morning. The same dose was administered at 10.30 A. M. The patient complained of general weakness and of numbness in the arm in which the injection was made. The pulse was slowed, but remained moderately strong. At four P. M. a watery passage occurred, after which none until twenty-seven hours after the injection, followed by stoppage of thirty-six hours; there was no vomiting. The patient was quite comfortable on the evening of the day of the injection, having passed the afternoon half asleep.

The third patient, a man of thirty-nine years, suffered from chronic dysentery, acquired in Africa. On the day before the medicine was used twenty-four bloody stools were counted by the attendants. On the day of the first injection (.001 eserine sulph.) twelve stools were passed of unchanged character. During the twenty-four hours following the second injection (.0015) there were five passages striped with blood, and during the next twenty-four hours, four bloody stools. In the two days following the third injection (.0015) seven stools were passed, of which three were accompanied by blood. During the four days following the fourth injection (.0015) the passages varied from one to six, some with and some without blood. Vomiting followed the first two injections only. The writer remarks that although the last case was not watched to its termination, and although he could not promise himself a perfect cure, the result was sufficiently marked to illustrate the beneficial action of the drug in this dose in limiting the weakening hemorrhages and albuminous stools. He also announces his intention of instituting shortly experiments on animals with regard to the contradictory action of the drug in large and small doses, a peculiarity already noticed in other medicaments.—*The Boston Medical and Surgical Journal.*

